# Development of a highly sensitive point‐of‐care test for African swine fever that combines EZ‐Fast DNA extraction with LAMP detection: Evaluation using naturally infected swine whole blood samples from Vietnam

**DOI:** 10.1002/vms3.1124

**Published:** 2023-04-03

**Authors:** Mai Thi Ngan, Huynh Thi My Le, Vu Xuan Dang, Trinh Thi Bich Ngoc, Le Van Phan, Nguyen Thi Hoa, Truong Quang Lam, Nguyen Thi Lan, Kosuke Notsu, Satoshi Sekiguchi, Yasuko Yamazaki, Wataru Yamazaki

**Affiliations:** ^1^ Faculty of Veterinary Medicine Vietnam National University of Agriculture Hanoi Vietnam; ^2^ Graduate School of Medicine and Veterinary Medicine University of Miyazaki Miyazaki Japan; ^3^ Department of Veterinary Science Faculty of Agriculture University of Miyazaki Miyazaki Japan; ^4^ Center for Animal Disease Control University of Miyazaki Miyazaki Japan; ^5^ Center for Southeast Asian Studies Kyoto University Kyoto Japan; ^6^ School of Public Health Kyoto University Kyoto Japan

**Keywords:** African swine fever, EZ‐Fast, field setting, loop‐mediated isothermal amplification, point‐of‐care test

## Abstract

**Background:**

While early detection and early containment are key to controlling the African swine fever (ASF) pandemic, the lack of practical testing methods for use in the field are a major barrier to achieving this feat.

**Objectives:**

To describe the development of a rapid and sensitive point‐of‐care test (POCT) for ASF, and its evaluation using swine whole blood samples for field settings.

**Methods:**

In total, 89 swine whole blood samples were collected from Vietnamese swine farms and were performed the POCT using a combination of crude DNA extraction and LAMP (loop‐mediated isothermal amplification) amplification.

**Results:**

The POCT enabled crude DNA to be extracted from swine whole blood samples within 10 min at extremely low cost and with relative ease. The entire POCT required a maximum of 50 min from the beginning of DNA extraction to final judgment. Compared to a conventional real‐time PCR detection, the POCT showed a 1 log reduction in detection sensitivity, but comparable diagnostic sensitivity of 100% (56/56) and diagnostic specificity of 100% (33/33). The POCT was quicker and easier to perform and did not require special equipment.

**Conclusions:**

This POCT is expected to facilitate early diagnosis and containment of ASF invasion into both regions in which it is endemic and eradicated.

## Graphical abstract

The developed POCT is quicker and easier to perform and does not require special equipment. We validated a sensitive POCT for ASF in swine whole blood samples for field settings.This POCT is expected to facilitate early diagnosis and containment of ASF invasion into both regions in which it is endemic and eradicated.

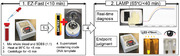

## 1 INTRODUCTION

The current African swine fever (ASF) pandemic, which began with the disease's 2007 invasion of Georgia from East Africa, is one of the greatest threats to the global livestock industry and food security today, making its control an urgent issue (Rowlands et al., 2008; Sauter‐Louis et al., 2021). ASF is a viral haemorrhagic disease of domestic and wild swine that is characterised by high fever, haemorrhages in the reticuloendothelial system, and a high mortality rate (Dixon et al., 2020; Galindo & Alonso, 2017; OIE, 2021a; Sánchez‐Vizcaíno et al., 2015). ASF is caused by the ASF virus (ASFV), which is the only virus of the family Asfarviridae comprising large (>200 nm) double stranded DNA (dsDNA) spherical viruses (Dixon et al., 2005). In sub‐Saharan Africa, ASFV is maintained through long‐term, inapparent infection of wild suids such as bush pigs (*Potamochoerus porcus*) and warthogs (*Phacochoerus africanus*), which become infected from the bites of the Argasid tick vector (*Ornithodoros* complex) (Dixon et al., 2020, 2019; Sauter‐Louis et al., 2021). The virus can also infect domesticated pigs and wild boars, where it can cause acute haemorrhagic fever, and subsequent high morbidity and mortality (Dixon et al., 2020, 2019; Galindo & Alonso, 2017; OIE, 2021a; Sánchez‐Vizcaíno et al., 2015).

Although ASF outbreaks were previously limited in sub‐Saharan Africa, the outbreak in 2007 in Georgia and the subsequent spread to neighbouring countries in the south Caucasus, Europe, and the Russian Federation has highlighted the threat of the transboundary spread of ASF (Rowlands et al., 2008; Sánchez‐Vizcaíno et al., 2015; Sauter‐Louis et al., 2021). Furthermore, the disease's invasion of China, the world's largest pig producer in 2018, and subsequently Southeast Asian nations including Vietnam has been devastating to the livestock industry and food security (Blome et al., 2020; Le et al., 2019; OIE, 2022; Wang et al., 2021; You et al., 2021; Zhou et al., 2018; Zhu et al., 2022). In 2021, ASF reached the Dominican Republic in the Caribbean, reaffirming the importance of quarantine systems in the Americas (Carriquiry et al., 2020; Gonzales et al., 2021; OIE, 2022). As a result of the expansion of ASF, pig industries have incurred significant economic losses in both endemic and ASF‐free countries (Carriquiry M, 2020; Mason‐D'Croz et al., 2020; Nguyen‐Thi et al., 2021; OIE, 2022; You et al., 2021).

Rapid and accurate diagnosis of ASFV is required to effectively control outbreaks, particularly to accurately identify asymptomatic carriers and/or first positive cases for early containment. Accurate differential diagnosis of classical swine fever (CSF), porcine pleuropneumonia caused by *Actinobacillus pleuropneumoniae*, heat stress, and poisoning, which have clinical manifestations similar to ASF, is also essential. However, in many countries, animal quarantine stations and regional livestock health centres, which are responsible for waterfront quarantine, do not have sufficient diagnostic facilities. Highly sensitive and rapid real‐time polymerase chain reaction (PCR) assays are widely used for routine diagnosis of ASF (Fernández‐Pinero et al., 2013; King et al., 2003; Trinh et al., 2021; Wang et al., 2020). However, due to the significant associated cost of equipment and reagents, these methods are impracticable for use in less equipped laboratories. Given these limitations, rapid, simple, sensitive, and cost‐effective methods for diagnosing ASF using simple isothermal amplification techniques such as loop‐mediated isothermal amplification (LAMP) and recombinase polymerase amplification (RPA) are urgently required (Ceruti et al., 2021; James et al., 2018; Wang et al., 2021; Wang et al., 2020).

Point‐of‐care tests (POCTs) such as immunochromatographic assays for antigen detection (Ag‐ICA) are the widely used test‐of‐choice for simple and rapid screening of transboundary animal diseases such as avian influenza and foot‐and‐mouth disease on farms (OIE, 2018, 2021b). However, no Ag‐ICA has yet been developed for ASF (OIE, 2021a). Although a POCT was recently developed for simple ASFV‐specific antibody detection (Zhu et al., 2022), the test requires preparation of serum and is not expected to be able to detect the virus in the early stages of infection when antibodies levels are insufficient for detection. Deployment of POCTs based on genetic tests for on‐site diagnosis in field settings is, therefore, an attractive option for early detection due to their high diagnostic performance (Ceruti et al., 2021; Shimetani, 2017; Tran et al., 2021; Wang et al., 2021). However, the conventional nucleic acid extraction step requires a commercial extraction kit and a high‐speed centrifuge. As an alternative, several studies have reported using a simple technique of diluting serum or whole blood samples more than 10‐fold with nuclease‐free water or lysis buffer to detect target pathogens (Ceruti et al., 2021; Tran et al., 2021; Wang et al., 2021; Zhou et al., 2022). The disadvantage, however, is a significant decrease in detection sensitivity. Meanwhile, only one preliminary study has been performed using just five whole blood samples without any dilution (Wang et al., 2021). Evaluation of whole blood samples from a large number of naturally infected individuals is needed.

To develop a more sophisticated POCT that maximises throughput, we previously combined a fast crude DNA extraction technique that uses sodium dodecyl benzenesulfonate (SDBS), which we named the EZ‐Fast kit, with a LAMP method for bovine leukaemia virus (BLV) diagnosis (Yamazaki et al., 2020). Here, we report the development of a POCT for on‐farm ASF diagnosis that combines this EZ‐Fast kit with newly designed LAMP primers. This POCT requires only a 2‐fold dilution of the whole blood sample and no special equipment, and allows for easy diagnosis through colour changes observable by the naked eye and fluorescence emission using a portable LED illuminator within 50 min. We evaluated the performance of the new POCT by comparing its diagnostic performance with that of a conventional method that combines a DNA extraction kit with real‐time PCR in 89 swine blood samples collected from ASF endemic farms in Vietnam.

## 2 MATERIALS AND METHODS

All laboratory work was performed at the (Anonymisation, name of research institution).

### 2.1 Primer development for the LAMP assay for specific ASFV detection

A new primer set was designed based on the p72 protein‐encoding region of the ASFV genome using Primer Explorer V5 software (https://primerexplorer.jp/lampv5/index.html). In silico analysis, conserved nucleotide sequences within the p72 protein‐encoding region were identified using multiple alignment of 216 ASFV sequences covering candidate sequences available from the DDBJ/EMBL/GenBank databases using BLAST (https://blast.ncbi.nlm.nih.gov/Blast.cgi). All primers were synthesised using a cartridge‐grade purification system by Hokkaido System Science Co. Ltd. (Sapporo, Japan). Details of the designed primers are shown in Table 1. In silico analysis, we confirmed that the target sequence, the p72 protein‐encoding region, was highly specific for ASFV and that the designed primers did not contain any nonspecific sequences that matched registered sequences other than that of ASFV. In advance, RNAs extracted from three isolates of classical swine fever virus (CSFV), which causes a disease with similar clinical signs in pigs were tested by the LAMP analysis described below and all were confirmed to be negative.

**TABLE 1:** LAMP primers designed for detection of the p72 protein‐encoding region of ASFV

Primer	Sequence (5'–3')	Gene location	Concentration (μmol/L)
ASF1‐F3	GGAAAAAGTCTCCGTACTG	106214–106232	0.2
ASF1‐B3	ATATGGCATCAGGAGGAG	106519–106502	0.2
ASF1‐FIP	TTGAGTCAAATCGAAGAAACACATACACTTTATTGTATTCAAACCCTA	106373–106349, 106287–106309	1.6
ASF1‐BIP	GCAAGTCTTGGGCCAAGATACTTTTTGTCTTATTGCTAACGATGG	106442–106461, 106501–106477	1.6
ASF1‐LF	GCATTTTAATGCACATTTTAAGCCTT	106344–106319	0.8
ASF1‐LB	AATCTTGTCGGCCTTCC	106461–106477	0.8

Locations correspond to the complete genome sequence of ASFV Georgia 2007/1 (GenBank Accession number NC_044959.2).

### 2.2 Clinical swine samples

From 3 June 2020 to 1 January 2022, 89 swine blood samples were collected from 38 ASF outbreak farms located in nine provinces in northern Vietnam (Table S1). Using evacuated blood collection tubes containing EDTA, whole blood samples were collected from pigs suspected of having symptoms of ASF in each farm, kept cool and delivered to our laboratory in Hanoi where they were stored at 4°C. DNA was immediately extracted from the swine blood samples and used for POCT and real‐time PCR analyses.

### 2.3 DNA extraction using the EZ‐Fast kit for POCT

**FIGURE 1:**
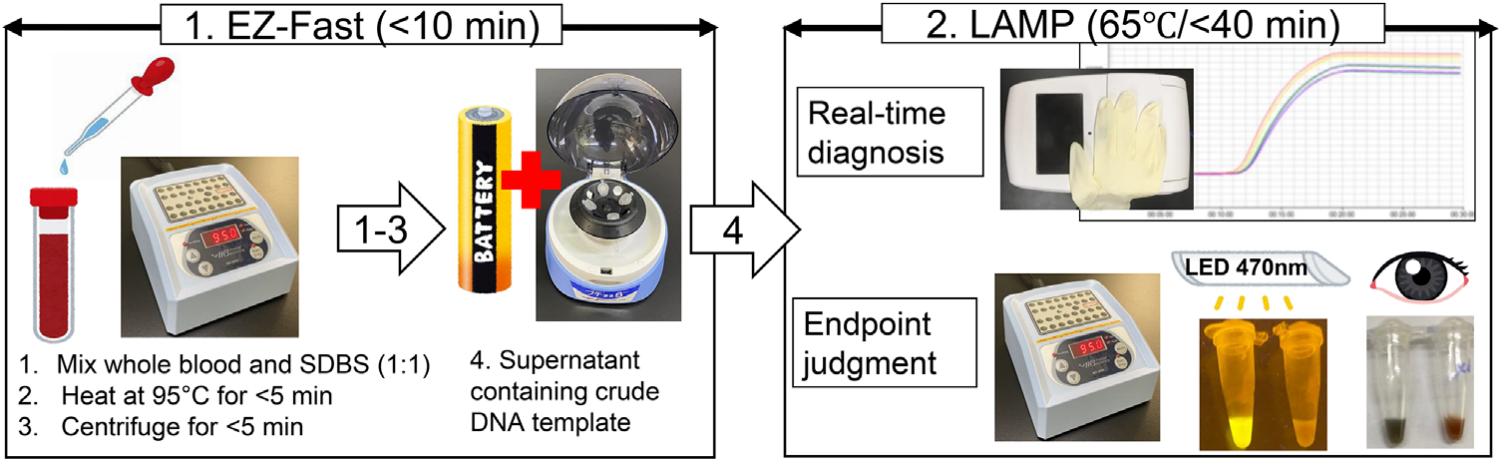
Concept of the POCT, which combines simple DNA extraction with EZ‐Fast and a heat block/portable battery‐powered LAMP device.

Crude DNA samples were prepared according to our previous report (Yamazaki et al., 2020). Namely, 500 μL of swine whole blood and 500 μL of 1% SDBS aqueous solution (Tokyo Chemical Industry Co., Tokyo, Japan) were mixed in a 1.5‐mL microcentrifuge tube by pipetting 5−10 times or vortexing for 5−10 s. The sample tube was placed in a portable heat block (Mini heating dry bath incubator, MD‐MINI, Major Science, Co. Ltd., Saratoga, CA, USA), incubated at 95°C for 5 min, and centrifuged in a dry‐cell battery‐powered table‐top centrifuge (Puchimaru 8, Wakenbtech, Co., Ltd., Kyoto, Japan) for 5 min at full speed (1260−2840 g) at room temperature. As a safeguard to avoid contamination by blood debris, a 20‐μL sample of the supernatant was transferred to a new 0.2‐mL microcentrifuge tube for LAMP analysis. Special attention was paid to retrieving the very top layer of the supernatant to avoid contamination with solid blood debris that may contain *Bst* polymerase inhibitors. The protocol for the DNA amplification step is conceptualised and shown in Figure 1.

### 2.4 LAMP assay for POCT

To make a 25‐μL reaction mixture, 12.5 μL of an in‐house LAMP reaction mixture with identical composition to that outlined in our previous reports (Yamazaki et al., 2020; Yashiki et al., 2019); 1.6 μmol/L each of inner primers FIP and BIP; 0.2 μmol/L each of outer primers F3 and B3; 0.8 μmol/L each of loop primers LF and LB; 1 μL of *Bst* polymerase 2.0 (8 units); and 1 μL of Colori‐Fluorescent Indicator (CFI) were added to a tube and the volume was made up to 20 μL with nuclease‐free water before adding 5 μL of the DNA template. For the 89 clinical swine whole blood samples, LAMP was performed using a simple heat block as described previously (Yamazaki et al., 2020) with the following modifications: the amplification was performed at 65°C for 40 min (for 30 min in the preliminary test), and the subsequent inactivation step at 98°C for 2 min.

For rapid diagnosis of ASF, the endpoint was determined with the naked eye. When the colour of the mixture of reagent and blood supernatant remained red or dark red, the result was interpreted as negative. On the other hand, a change to green or dark green was defined as positive. In addition, if the mixture fluoresced at 470 nm under illumination with a portable blue light (SAFEVIEW‐MINI2, Cleaver Scientific Ltd., United Kingdom), the result was also determined to be positive. To determine the limit of detection (LOD), the LightCycler*^®^* 96 System (Roche, Basel, Switzerland) was used for LAMP amplification and time‐to‐positivity (Tp) determination. Details of the protocol are described below.

### 2.5 Conventional DNA extraction

Total DNA was extracted from 200 μL of each of the 89 whole blood samples using the QIAamp DNA Mini Kit (Qiagen, Maryland, USA). Total DNA was eluted in 50 μL of nuclease‐free water and stored at −80°C until real‐time PCR analysis.

### 2.6 Conventional real‐time PCR assay

Using the LightCycler*^®^* 96 System (Roche), real‐time PCR was performed using a commercial real‐time PCR kit (Median Diagnostics Inc., Korea) according to the manufacturer's instructions and previous reports (Bokyu et al., 2019; Tran et al., 2021), which showed equivalent diagnostic performance to the protocol described by Fernández‐Pinero et al. (2013) listed in the OIE manual (2021b). Briefly, each 20 μL PCR reaction contained 5 μL 4X Oligo Mix and 10 μL 2X qPCR Master Mix to amplify 5 μL of template DNA. Reactions were performed in duplicate using the following cycling conditions: 10 min at 95°C, 40 cycles at 95°C 15 s and 58°C 60 s, and fluorescence was acquired in the 6‐carboxyfluorescein (FAM) channel at the end of each PCR cycle. Following amplification, threshold cycle (C_T_) values were assigned and the mean C_T_ value from duplicate samples was adopted for analysis. This study adopted a cut‐off C_T_ value of 35.0, with samples determined to be positive when the mean gave a C_T_ value of <35.0. Conventional column kit DNA extraction combined with the real‐time PCR assay was used as the gold standard against which to evaluate the POCT.

### 2.7 Comparison of LOD between the POCT and conventional assays using swine whole blood samples

The LOD of the POCT was assessed in the LightCycler*^®^* 96 System (Roche) using an ASFV‐positive whole blood sample (no. 89; Table S1). Real‐time LAMP was performed as follows: amplification was performed at 65°C for 60 min in the FAM channel (excitation wavelength 470 nm, emission wavelength 514 nm), followed by an inactivation step at 98°C for 2 min. A positive LAMP reaction was indicated by an exponential increase in fluorescence, and Tp was defined as the time needed for the amplification curve to reach an automatically set threshold value. Samples in which fluorescence values reached the threshold within 60 min were considered positive. The ASFV‐positive whole blood sample was serially diluted 10‐fold with a pool of ASFV‐negative samples (nos. 78, 81, 82, 87 and 88), and the POCT and conventional real‐time PCR assays were performed as described above and analysed in duplicate. The LOD was defined as the highest dilution at which both replicates tested positive.

## 3 RESULTS

The results of the POCT were perfectly consistent with those of the conventional real‐time PCR method. As shown in Table 2, the POCT achieved a diagnostic sensitivity and specificity of 100% (56/56) and 100% (33/33), respectively. Namely, using the conventional method, all 33 negative samples were determined to be negative, and all 56 positive samples were determined to be positive. No nonspecific false positives and false negatives were obtained with the POCT. Unlike the conventional column kit DNA extraction, which required around 30 min and several tedious steps, the EZ‐Fast kit required only 10 min to extract crude DNA from swine whole blood samples and cost less than $0.1 (USD) per sample (Figure 1), similar to our previous report using bovine whole blood samples (Yamazaki et al., 2020). In a preliminary test, when the amplification was attempted 30 min determination, all samples with C_T_ values up to 27.24 in the conventional method were also positive in the POCT, whereas two positive samples with C_T_ values of 31.70 (no. 15) and 32.30 (no. 17) were false negatives in the POCT (Table S1).

**TABLE 2:** Diagnostic performance of LAMP using automatically and EZ‐Fast‐extracted DNA

	Conventional method (real‐time PCR with column kit‐extracted DNA)	
POCT (EZ‐Fast DNA extraction combined with LAMP)	Positive	Negative
Positive	56	0
Negative	0	33

Diagnostic sensitivity, 100% (56/56); diagnostic specificity, 100% (33/33).

**FIGURE 2:**
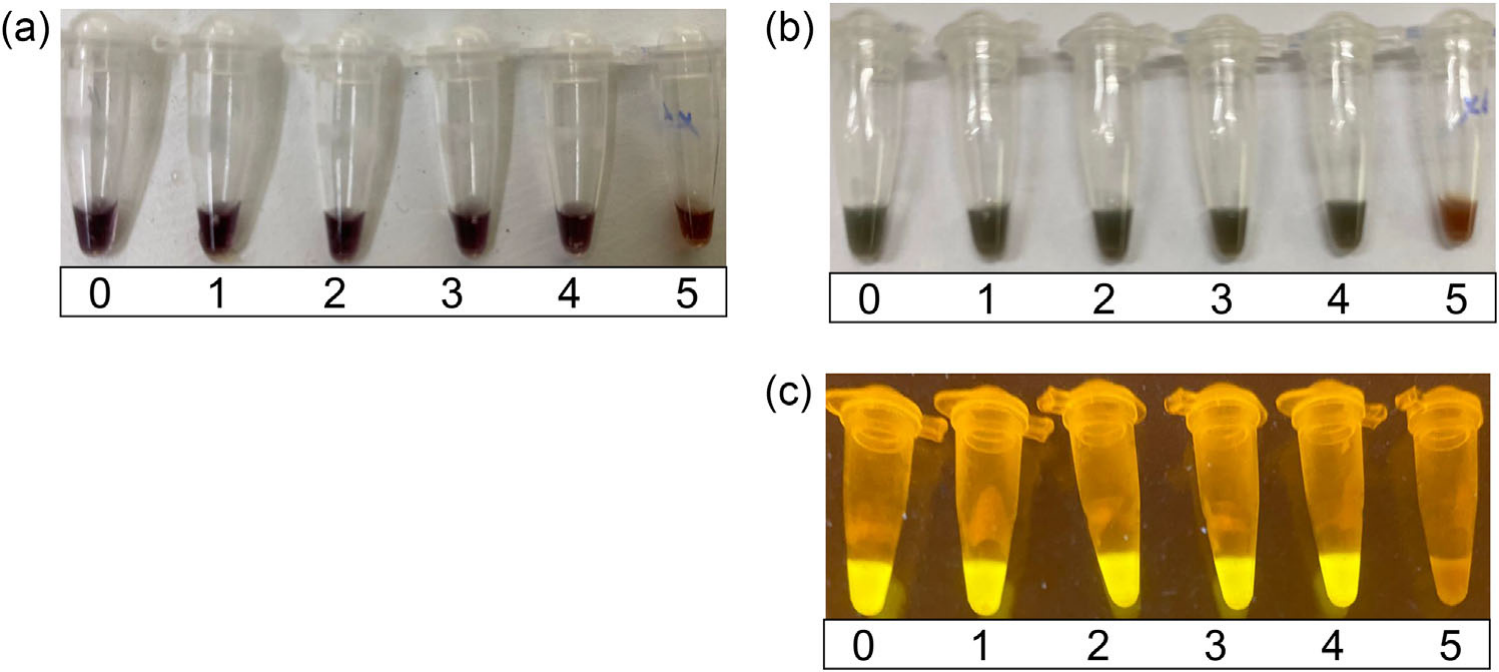
Limit of detection of the POCT for ASF detection. (A) Before amplification. (B) Changes after amplification can be detected as a colour shift from red to green by the naked eye. (C) Changes can also be observed under LED light. 0, Original ASFV‐positive blood sample; 1–5, 10‐fold serial dilutions of the ASFV‐positive sample in pooled negative blood samples.

Despite the presence of residual blood pigment, diagnosis results obtained with the naked eye and under LED illumination with a portable blue light following the LAMP assay and DNA extraction using the EZ‐Fast kit matched those obtained with fluorescent real‐time LAMP detection using a bench‐top real‐time PCR machine (Figure 2). As shown in Figure 2 and Table 3, the LOD using the POCT was 10‐fold lower than that using the conventional method. While the conventional method using column kit‐extracted DNA showed a positive result in samples diluted up to 10^5^‐fold, the POCT using EZ‐Fast DNA extraction yielded positive results in those diluted up to 10^4^‐fold and a negative result at the 10^5^‐fold dilution, as expected.

**TABLE 3:** Comparison of the limit of detection (LOD) between the conventional real‐time PCR and the POCT for ASF detection

Dilutions	0	1	2	3	4	5	6
Conventional (C_T_)	19.28	20.76	24.30	27.86	31.11	34.99	No. C_T_
POCT (Tp)	21:46	22:00	25:44	26:14	29:16	No. Tp	No. Tp

0, Original ASFV‐positive blood sample; 1–6, 10‐fold serial dilutions of the ASFV‐positive sample in pooled negative blood samples.

C_T_, threshold cycle; Tp, time to positivity.

## 4 DISCUSSION

The current ASF pandemic and the resulting financial damage highlight the need for early containment and control of ASF throughout the world (Carriquiry, 2020; OIE, 2022; You et al., 2021; Zhou et al., 2018). On‐site diagnosis in the field using portable molecular diagnostic devices can facilitate the containment of outbreaks of both veterinary and human viral diseases (Bernstein et al., 2022; Ceruti et al., 2021; Kurosaki et al., 2016; Semper et al., 2016; Tran et al., 2021; Wang et al., 2021; Yamazaki et al., 2021, 2020).

By combining the EZ‐Fast kit with LAMP, we successfully and rapidly detected BLV proviral DNA in bovine whole blood with high sensitivity in our previous study (Yamazaki et al., 2020). In the present study, we showed that the same method can be used to detect ASFV in swine whole blood. Notably, crude DNA extraction using the EZ‐Fast kit from swine whole blood samples required only 10 min. As expected, simply heating samples at 95°C for 5 min followed by a 5‐min centrifugation using a portable table‐top centrifuge at full speed (1260−2840 g) was sufficient to obtain crude DNA suitable for LAMP amplification, despite significant contamination with swine blood components. Conventional DNA extraction using a column kit required around 30 min, and the subsequent real‐time PCR method took another 90 min to provide a result. In contrast, the LAMP method provided results within 40 min for samples yielding low real‐time PCR C_T_ values (Tables 3 and S1 and Figure 2). If rapidity is compromised, the combination of the EZ‐Fast kit with a real‐time PCR reagent highly resistant to inhibitors and a portable real‐time PCR device has the potential to lead to the development of even more sensitive POCTs (Howson et al., 2018; Yamazaki et al., 2020).

Tables 2 and S1 compare the results of the performance evaluation of the POCT and conventional method. To examine these two weak positive samples more precisely, real‐time LAMP was performed at 65°C for 60 min using SDBS‐extracted DNAs, both of which turned positive at 33 min 02 s for no. 15, and at 38 min 46 s for no. 17, respectively (data not shown). Namely, the POCT developed in this study resolved these two ambiguous results by employing a 40‐min amplification time instead of the 30‐min amplification attempted in the preliminary test. As shown in Table 3 and Figure 2, the results of the LOD comparison demonstrated that the POCT for 40 min amplification was 10 times less sensitive for detecting the ASFV than the conventional method. Therefore, we assumed that these two samples (nos. 15 and 17) were near the LOD of the POCT.

Various POCTs have been developed for early diagnosis of ASF in serum (Ceruti et al., 2021; Tran et al., 2021; Wang et al., 2020) or whole blood (Ceruti et al., 2021; Wang et al., 2021). Due to the tropism of ASFV‐infected monocytes and macrophages, the virus readily adsorbs onto erythrocytes (OIE, 2021b), leading ASFV to be more abundant in whole blood than in serum. For this reason, we believe that whole blood, which contains erythrocytes, is a better material to use for ASFV detection than serum. Furthermore, unlike serum, whole blood does not require high‐speed centrifugation for preparation in the field. Therefore, we propose that whole blood is the more appropriate sample for highly sensitive ASF diagnosis in field settings. While previous a study has only preliminarily validated POCT based on different principles for crude DNA extraction using whole blood on a limited scale of five samples (Wang et al., 2021), we analysed 89 whole blood samples for a robust evaluation. A recently developed POCT based on the RPA method for ASFV detection is also an attractive option for rapid and sensitive ASF diagnosis, adopting a 20‐min incubation of a 1:1 whole blood and lysis buffer mixture at 70°C for crude DNA extraction. However, it is less convenient and sensitive than the POCT reported in the present study as it necessitates the use of a special fluorometer and commercial reagent, as well as 10‐fold dilution of the crude DNA template in nuclease‐free water (Ceruti et al., 2021).

Despite several advantages, however, the POCT reported in this study also has some limitations. Because the POCT uses SDBS to dilute swine whole blood 2‐fold, the LOD is nearly 10‐fold lower than that of the existing DNA extraction kit, which concentrates the DNA in the sample to around 4‐fold. Furthermore, the LAMP method has up to 10‐fold lower LOD than the real‐time PCR method (Yamazaki et al., 2020). Thus, while the POCT shows sufficient diagnostic performance for individuals with high viral load, it may be prone to producing false‐negative results for those with low viral load. Therefore, samples determined to be negative by the POCT should be carefully evaluated in the laboratory using a combination of column kit DNA extraction and highly sensitive real‐time PCR methods. Further, while the combination of the EZ‐Fast kit with LAMP enables the CFI to be visualised by the naked eye through changes in colour, discrimination of differences are hindered partly by the presence of residual haemoglobin. Therefore, determining the endpoint using portable LED illuminators or portable LAMP devices such as Genie III for real‐time monitoring should be prioritised as a safeguard (Hayashida et al., 2015). Additionally, the POCT is qualitative, and therefore unable to provide a quantitative analysis of the viral load.

As conceptualised in Figure 1, even without a portable real‐time measurement device, a simple heat block allows endpoint analysis to be performed using the naked eye or LED illumination within 40 min. Such simple detection methods with the POCT will make DNA preparation quicker (within 10 min) and easier, making the POCT suitable for use in the field. Current routine diagnostic systems require collected whole blood samples to be transported to the laboratory due to the lack of a highly sensitive POCT. Eliminating this time lag will minimise the damage caused by ASF, providing a clear benefit to all stakeholders, including farmers, consumers, veterinarians, and government agencies.

In conclusion, the POCT developed in this study, which combines the EZ‐Fast kit based on SDBS for crude DNA extraction with LAMP detection, is a rapid, simple and practical method with potential application in on‐site diagnosis, making it a valuable tool for containing and managing ASF outbreaks.

## AUTHOR CONTRIBUTIONS

All authors contributed to the study's conception and design. Conceptualisation: MTN, HTML, NTL, SS, YY, WY. Primer design and optimisation: KN, YY, WY. Investigation: VND, TTBN, LVP, NTH, TQL. Data analysis: MTN, WY. Writing – original draft preparation: MTN, WY. Writing – review and editing: MTN, YY, WY. Funding acquisition: MTN, NTL, YY, WY. All authors read and approved the final manuscript.

## FUNDING

This research was supported by JSPS KAKENHI under the grant number JP22K05950, JP22KK0097, and by Japan‐ASEAN Transdisciplinary Studies, Center for Southeast Asian Studies, Kyoto University.

## CONFLICT OF INTEREST STATEMENT

The concept behind EZ‐Fast is the subject of a pending patent by YY and WY in Japan (Japanese Patent Application nos. 2019‐193125 and 2020‐98951). The authors declare that they have no other conflicts of interest.

## ETHICS STATEMENT

The authors confirm that the ethical policies of the journal, as noted on the journal's author guideline page, have been adhered to. Samples used in this study were those submitted to the Vietnam National University of Agriculture, Hanoi, Vietnam, for ASFV diagnosis. Ethical approval was not required.

### PEER REVIEW

The peer review history for this article is available at https://publons.com/publon/10.1002/vms3.1124.

## Supporting information



Supporting Information

## Data Availability

Data available in article supplementary material.
